# Indwelling ureteric stents: Patterns of use and nomenclature

**DOI:** 10.1080/2090598X.2020.1761675

**Published:** 2020-05-19

**Authors:** Joon Yau Leong, James E. Steward, Kelly A. Healy, Scott G. Hubosky, Demetrius H. Bagley

**Affiliations:** a Department of Urology, Thomas Jefferson University, Philadelphia, PA, USA; b Department of Urology, Columbia University Medical Center, New York Presbyterian Hospital, New York, NY, USA

**Keywords:** Ureteric, stent, indwelling, nomenclature, double pigtail, Double J

## Abstract

**Objectives**: To evaluate ureteric stenting practice patterns amongst a range of academic and community urologists, and to examine the nomenclature used to identify an indwelling ureteric stent from both our questionnaire and from a review of the literature.

**Subjects and methods**: A 16-question, peer-reviewed online survey was distributed to members of the Mid-Atlantic American Urological Association. Responses were collected over a 1-month period. Questions included demographics, ureteric stenting practice patterns, and utilization of stenting nomenclature. Inappropriate use of nomenclature was defined as a mismatch between the visually depicted stents and the written description amongst urologists. Trends in ureteric stenting and nomenclature usage were tabulated and analyzed.

**Results**: Of 863 members, 105 (12.2%) responded to the survey. There was a wide variety of practice settings, with the single-specialty group (44.2%) and academic/university (27.9%) being the two most common. Most providers used both cystoscopy and fluoroscopy to place stents (87.5%) as compared to fluoroscopy alone (12.5%). Most urologists (63.5%) removed stents with cystoscopy as compared to using a stent string (36.5%). While about half (51.0%) of the respondents left stents *in situ* for ≤3 months, many respondents (43.3%) felt comfortable with maximum dwell times of up to 6 months. The most commonly placed stent was the double pigtail stent (80.8%). However, most respondents inappropriately described this stent design as a Double J stent (72.1%). In the recent literature, 80% of articles clearly defined as using double pigtail stents, incorrectly identified their stent as a ‘Double J’.

**Conclusions**: Variations in ureteric stenting practice patterns exist amongst community and academic urologists. Although most urologists utilize double pigtail ureteric stents, the majority inaccurately identified this stent design as a Double J. We propose use of the term ‘indwelling ureteric stent’ (IUS) unless describing any specific stent design.

## Introduction

Ureteric stent placement is a common urological procedure. Indications for stent placement include relief of obstruction, post-ureteroscopy placement for obstruction prevention, ureteric identification/designation, and as a splint after ureteric repair or operation. Associated risks and complications of ureteric stenting include stent migration, encrustation, injury to the ureter, loss of patency, infection, and retained stent. The objectives of the present study were to explore trends of ureteric stenting amongst a diverse group of urologists. We also aimed to investigate the specific terminology used to describe ureteric stents amongst urologists, as well as within the current literature. Finally, we propose the use of the generic term ‘indwelling ureteric stent’ (IUS) unless describing the specific stent design.

## Subjects and methods

A 16-question, online survey was developed using SurveyMonkey**®**. It included questions pertaining to provider demographic data, background in training, current practice setting, frequency of stent placement, stent type, method of stent placement, and postoperative management. The survey was peer-reviewed and approved by the Mid-Atlantic AUA (MA-AUA) and sent to all members via e-mail. The study period was 1 month. The e-mail was distributed in one ‘blast’ with no follow-up reminders per the organization’s guidelines. Inappropriate use of nomenclature was defined as a mismatch between the visually depicted stent and the written description used in urological practice. Results were then tabulated to assess trends in ureteric stenting and nomenclature usage.

Additionally, a PubMed database search was performed using the phrase ‘ureteral stent’. A total of 50 studies were identified and evaluated in March 2020. The terminology used to describe the stent was noted, as was any visual depiction or description of the physical characteristics of the stents.

## Results

Of 863 members, 105 (12.2%) responded to the survey. Of these, one respondent did not routinely place stents within their practice and was excluded from the study. Table 1 provides the demographic data and practice patterns based on survey responses. The largest group of respondents were aged 45–54 years (28.8%), followed by 55–64 years (24.0%), and 35–44 years (20.2%). The majority were male (88.5%) and over two-thirds (72.2%) had been in practice for >10 years. Practitioners in single-specialty groups comprised the largest number of respondents at 44.2%, followed by full-time academic staff (27.9%), multi-specialty group practice (11.5%), and solo practice (11.5%). Ureteric stenting was a common practice amongst respondents with nearly all responders (97.1%) reporting placing stents at least several times per month and nearly two-thirds (64.4%) placing stents at least several times per week.

**Table 1.** Demographic data and practice patterns

Survey questions	Survey responses, *n* (%)
Age range, years	
25–34	14 (13.5)
35–44	21 (20.2)
45–54	30 (28.8)
55–64	25 (24.0)
65–74	13 (12.5)
≥75	1 (1.0)
Gender	
Male	92 (88.5)
Female	12 (11.5)
Years in practice	
<5	10 (9.6)
5–10	19 (18.3)
10–20	27 (26.0)
>20	48 (46.2)
Practice type	
Academic	29 (27.9)
Single-specialty group	46 (44.2)
Multi-specialty group	12 (11.5)
Solo practice	12 (11.5)
Other	5 (4.8)
Endourology fellowship completed	
Yes	10 (9.6)
No	94 (90.4)
Member of Endourological Society	
Yes	23 (22.1)
No	81 (77.9)
Frequency of stent placement	
Almost daily	7 (6.7)
Several times per week	60 (57.7)
Several times per month	34 (32.7)
Several times per year	3 (2.9)
Description of stent in operative note	
Double J	75 (72.1)
Double pigtail	23 (22.1)
Others	6 (5.8)
String left on stent	
Yes	38 (36.5)
No	66 (63.5)
Use of fluoroscopy or cystoscopy during stent placement	
Fluoroscopy alone	13 (12.5)
Cystoscopy and fluoroscopy	91 (87.5)
Stent diameter, F	
4.7	14 (13.5)
6	87 (83.7)
7	0 (0.0)
8	0 (0.0)
Other	3 (2.9)
Stent length, cm	
20	1 (1.0)
22	3 (2.9)
24	52 (50.0)
26	26 (25.0)
28	1 (1.0)
Multi-length	16 (15.4)
Other	5 (4.8)
Maximum indwelling stent time, months	
1	3 (2.9)
3	50 (48.1)
6	45 (43.3)
12	2 (1.9)
Other	4 (3.8)
Aware of the cost of most commonly placed stent	
Yes	61 (58.7)
No	43 (41.3)
Medications used for stent-related symptoms	
α-blocker	19 (18.3)
Anticholinergic	15 (14.4)
Phenazopyridine	8 (7.7)
Combination of ≥2	57 (54.8)
None	5 (4.8)

More than one-third (36.5%) reported leaving a string on the stent for later removal. The majority used a combination of cystoscopy and fluoroscopy (87.5%) when placing the stent, while the remainder used fluoroscopy alone. The most commonly used stent diameter was 6 F (83.7%); while the most commonly placed stent length was 24 cm (50.0%).

Participants were also asked ‘what was the maximum duration that they would feel comfortable leaving an indwelling stent, whenever indicated or necessary’. Nearly half (48.1%) felt comfortable leaving it for a maximum of 3 months, while 43.3% felt comfortable leaving the stent in for up to 6 months, depending on the type of stent or whenever indicated, e.g. management of ureteric strictures. Most urologists (58.7%) knew the cost of the stent they usually place. Almost all providers (95.2%) prescribed an α-blocker (18.3%), anti-cholinergic (14.4%), phenazopyridine (7.7%), or a combination (54.8%) of these medications for stent-related symptoms.

**Figure 1.**
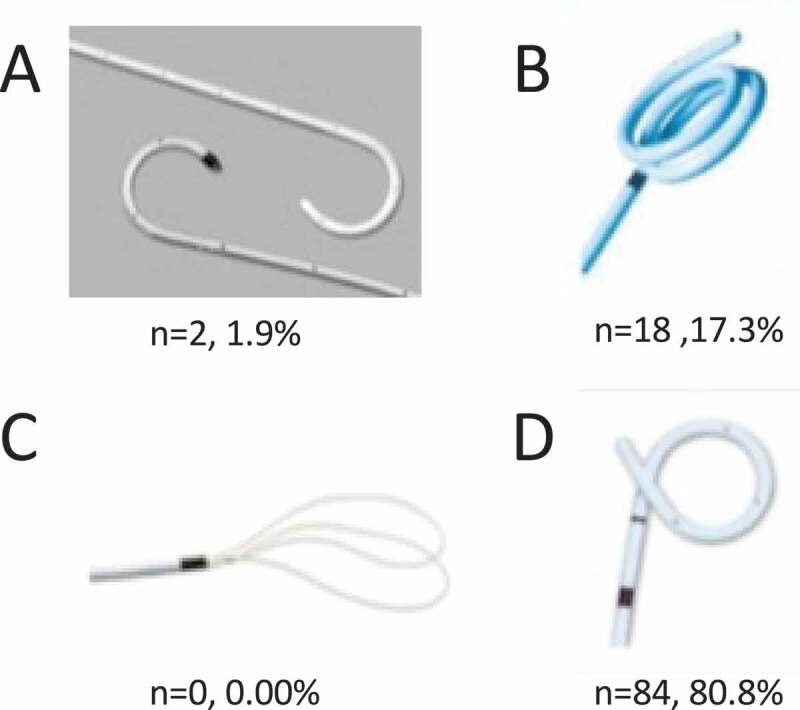
Images of four different stents and their respective survey response rate. Survey participants were blinded to stent nomenclature. (a) Double J stent, (b) Multi-length stent, (c) Loop stent, (d) Double pigtail stent

Participants were also asked to identify, from a picture of four different stents, the stent they most commonly placed (Figure 1). The majority (80.8%) chose the double pigtail stent. The multi-length stent was the next most commonly placed stent (17.3%). Double J stents were used by 1.9% of participants. Interestingly, the majority (72.1%) described stents as a Double J stent during dictation, while only 22.1% described it as a double pigtail stent.

**Figure 2.**
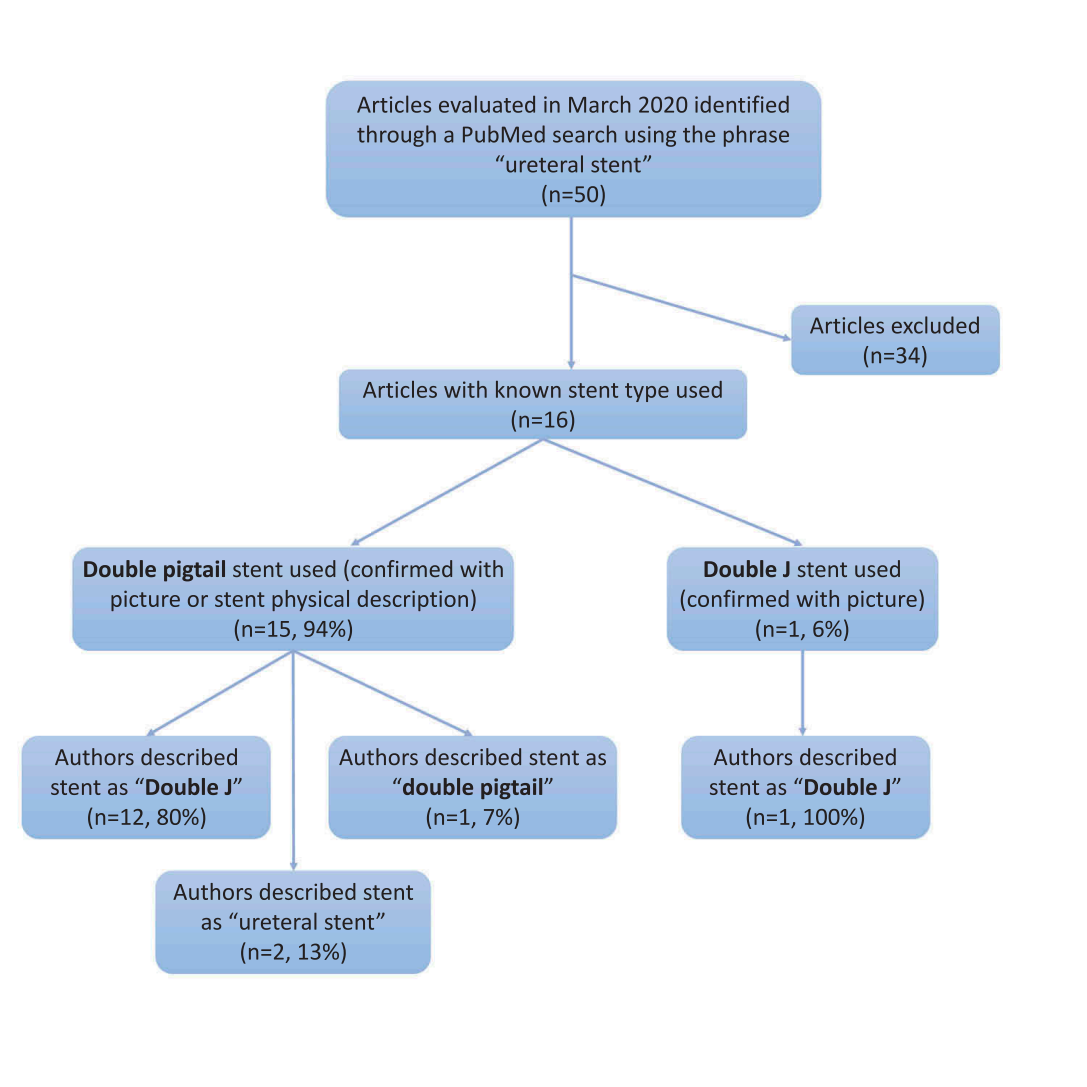
Flowchart of the PubMed literature review

An online PubMed search was performed using the keywords ‘ureteral stent’ and 50 articles were identified in March 2020. In all, 16 articles describe stent use, either as a picture or a description of the physical characteristics. Of these 16 articles, 15 (94%) used double pigtail stents. Of the articles using double pigtail stents, 80% described the stent as a ‘Double J’ (Figure 2).

## Discussion

Ureteric stents are utilized frequently by urologists. However, deployment of these stents often gives rise to side-effects that may affect a patient physically and psychologically. As such, ever since its inception in the late 1960s, the applications, indications, designs, features and models of ureteric stent have evolved over the decades, all with a common goal aimed at improving its durability, effectiveness and tolerability [1]. In 1978, Hepperlen et al. [2] first described the use of a ‘pigtail’ at one end of a ureteric stent, which describes a full 360°curl at the end of the stent. Later that year, Finney [3] described the Double J ureteric stent, which included a 180 ° J curve at each end to prevent distal and proximal migration. The double pigtail ureteric stent was subsequently described in 1979, which resembles today’s most commonly placed stent [4].

The Double J stent is a specific design with a 180 ° bend at each end and a closed proximal tip. It was first patented in the USA in 1980 (#4212304), and the name ‘Double J’ was trademarked in the same year by the Medical Engineering Corporation (Serial No. 73265596). The materials used to manufacture these stents initially included silicone for the Double J and polyethylene for the double pigtail. This has largely been replaced by polyurethane and other copolymers for increased flexibility and biocompatibility.

Over 70% of respondents identified the Double J stent (180°curl) use in their operative note. However, the majority (81%) chose the picture of the double pigtail stent (360°curl) when asked which stent most resembles their stent choice. This suggests that most urologists mislabel or misidentify the double pigtail stent for the Double J. This finding was consistent with a review of recent literature in which 80% of publications that used a double pigtail stent described their stents as a Double J. It is important to note that Double J stents may have a higher chance of proximal migration in comparison to the double pigtail stent, as a distal curl of <180 ° has been shown to be associated with a significantly higher risk of proximal stent migration.

In the discipline of endourology, specific nomenclature promotes clear, succinct communication and ultimately, quality patient care. For example, there is an important difference between *retrograde* and *antegrade* endoscopic approaches. Similarly, ‘pigtail’ and ‘J’ both describe two structurally different stents. In our survey and brief review of the literature, we consistently found that 80% of urologists used incorrect nomenclature when referring to a double pigtail ureteric stent.

The reason for this occurrence is unclear, although speculations can be made. Branding may have played a role. The term Double J in relation to the ureteric stent can be likened to Kleenex® as it relates to facial tissues. Or perhaps, as the clinically superior ‘pigtails’ supplanted the ‘Double Js’, urologists and stent manufacturers preferred fewer syllables with ‘dou-ble-J’. Additionally, as there was an almost universal adoption of pigtail stents by endourologists, the incorrect use of clinical nomenclature did not carry with it an overall negative clinical outcome during the transition. Nonetheless, continual misuse of the term Double J serves to underappreciate the importance of the ‘double pigtail’ and ignores the ingenuity and innovation that was required to create its superior design.

We propose using the generic term ‘indwelling ureteric stent’ with the acronym being ‘IUS’. This would serve as a universal term to describe Double J, double pigtail, and other self-retaining stent types, and it would help differentiate between ureteric catheters that are internal only vs. those that extend outside the body. This concept is supported by Current Procedural Terminology (CPT) codes, which distinguish between the two drainage designs. The code 52332 represents ‘insertion of indwelling ureteric stent’, while the code 52005 represents ‘ureteric catheterisation’. If one needs to describe the specific stent design, the correct terminology should be used (i.e. double pigtail stent, Double J stent, loop stent, multi-length stent, etc.). Appropriate descriptions of these devices would foster better communication amongst providers and could lead to better outcomes, as functional differences may exist amongst stent types. Looking forward, as novel stents and designs are developed, it would be even more important to use correct terminology when describing ureteric stents in operative notes or medical reports to ensure accuracy and to avoid any confusion or miscommunication.

Another goal of the study was to assess current stenting practice patterns amongst urologists. The most typical diameter of stents placed by survey respondents was 6 F (83.7%), with the 4.7 F diameter stent being the second most common (13.5%). Erturk et al. [5] randomized 46 patients undergoing ureteroscopy for stone disease to have a 4.7-F or 6-F stent placed for 1 week postoperatively. They found no difference in patient-reported pain or irritative symptoms. However, there was a significantly increased percentage of stents that migrated distally in the 4.7-F stent group. They recommended placement of 6-F stents based on these findings. Yet another recent study by Nestler et al. [6] in 2019 also showed that while the success of ureteroscopy is not compromised by a smaller stent diameter, thinner stents significantly decreases the risk of discomfort and pain postoperatively. Hence, ureteric stents with small diameters should be preferred.

Stent length, unlike stent diameter, has been shown to correlate with degree of stent-related symptoms. Choosing the correct length stent has the potential to reduce these symptoms. In a study of 60 patients with stents for 1 week after stone surgery, increased symptom severity positively correlated with stents crossing the midline and stents with an incomplete distal curl [7]. In a study of 120 patients, urgency, dysuria, and overall decreased quality of life were associated with longer stents, which was defined as the proximal end of the stent terminating in an upper calyx, while the distal end crosses the midline [8]. This was consistently true in a study by Inn et al. [9], which found that distal placement of a ureteric stent that crosses the midline to the contralateral site of the bladder significantly increases the risk of urinary irritative symptoms and body pain. Along with reduction of stent-related symptoms, proper stent length is also associated with decreased proximal migration rate [10]. In a study of 156 patients, measurements of the ureter made on CT (from the renal vein to the uretero-vesical junction) most accurately predicted ureteric length when measured with a ruled-5-F ureteric catheter. This was in comparison to measurements made with body height, body surface area, and linear distance on intravenous pyelograms (IVP) [11]. As such, this may be a reliable method to estimate ureteric lengths and prepare for the appropriate stent length preoperatively to minimize postoperative stent-related discomfort.

Finally, while there have been many studies evaluating the effects of ureteric stent diameter and stent length on patient experiences and stent-related symptoms, there have not been many head-to-head studies comparing these outcomes to ureteric stents constructed from different materials. Yet, in a prospective study with 50 patients conducted by Gadzhiev et al. [12], the authors compared the tolerability of silicone stents to polyurethane stents. Notably, they found that compared to the polyurethane stents, silicone ureteric stents were associated with a much lower reported pain score and pain intensity. Other variables such as the overactive bladder awareness tool, difficulty of stent placements and complications of haematuria or stent encrustation were not significantly different between the two materials. As the literature comparing stent material to patient outcomes remain scarce, future studies are necessary to further evaluate this topic at hand to better improve patient’s quality of life.

Up to 80% of patients with ureteric stents will experience stent-related symptoms, such as urgency, frequency, dysuria, suprapubic pain, and flank pain. In our survey, >95% of urologists prescribed an α-blocker, anti-cholinergic, phenazopyridine, or a combination of these medications for stent symptoms. A meta-analysis of 12 randomized controlled trials verified that α-blockers are associated with improvement in ureteric stent-related symptoms [13]. Lee et al. [14] found a lower total symptom score, as well as urgency, urge incontinence, flank pain, abdominal pain, urethral pain, and haematuria in patients given solifenacin after uncomplicated ureteroscopy and stent placement when compared to a control group. Similar results were seen with patients randomized to tolterodine vs placebo, with tolterodine demonstrating decreased postoperative symptoms [15].

Almost two-thirds of participants removed the stent string prior to placement. With increasing concern for harm to patients, cost to the healthcare systems and liability risk from retained stents, it will be interesting to see if the number of providers leaving a string on the stent increases in the future. A forgotten ureteric stent submits the patient to otherwise unnecessary imaging, procedures, and possible loss of renal function or even loss of an entire kidney. In a cost-effectiveness study of 27 patients with forgotten stents, Sancaktutar et al. [16] determined the economic cost is seven times higher than the cost of a timely removal. In a review of 493 urological cases in the UK, in which a total of >20 million pounds in indemnity claims were paid, retained ureteric stents were the most common postoperative-related claim [17].

The majority of urologists in our present survey felt comfortable leaving the stent *in situ* for ≤3 months. Prolonged dwell time is the most important risk factor in the development of encrustation. In a study of 330 ureteric stents, the encrustation rates were 26.8% at <6 weeks, 56.9% at 6–12 weeks, and 75.9% at >12 weeks [18]. Most stents need to be replaced at least every 3 months, but some are approved for up to 6–12 months. Additionally, most respondents prefer the use of both cystoscopy and fluoroscopy during stent placement. While the cystoscopy procedure increases operative time, potential benefits include decreased radiation exposure to patients and providers, as fewer fluoroscopy images are necessary. Potential risks of additional cystoscopy include urethral trauma and displacement of the safety wire.

Limitations of our present study include the limited geographical distribution of the survey, which was restricted to the Mid-Atlantic region of the USA. This group was chosen, as opposed to the Endourological Society for example, because it provided a broader representation of urologists in practice. The survey response rate was only 12.2%, and this may have been limited by the one-blast e-mail allowance. However, this rate is consistent with the typical rate of external surveys [19,20]. The survey was limited to those with e-mail access and those who were members of the organization. Additionally, survey data were also limited by provider recall bias. Future directions include exploring temporal trends in these practice patterns, especially if and when new stent technology, including drug-eluting and biodegradable stents, becomes more commonplace.

## Conclusion

In conclusion, stent placement is a common practice amongst urologists in a variety of settings. However, variations in ureteric stenting practice patterns exist amongst community and academic urologists, as evidenced by our present survey responses. The most common stent diameter and length placed was 6 F and 24 cm, respectively. Most urologists feel comfortable leaving a stent *in situ* for ≤3 months. Almost all urologists who completed the survey prescribe an α-blocker, anti-cholinergic, phenazopyridine, or a combination for stent-related symptoms. In terms of stent nomenclature, although most urologists utilize the double pigtail ureteric stent, the majority often mislabel and misidentify the double pigtail for a Double J, even though the terms describe two structurally distinct stents. Our review of the current literature also revealed a similar error rate. We propose the use of the generic term ‘indwelling ureteric stent’ (IUS) unless describing the specific stent design.
